# Mesothelin: An Immunotherapeutic Target beyond Solid Tumors

**DOI:** 10.3390/cancers14061550

**Published:** 2022-03-18

**Authors:** Joshua R. Faust, Darcy Hamill, Edward Anders Kolb, Anilkumar Gopalakrishnapillai, Sonali P. Barwe

**Affiliations:** Nemours Centers for Childhood Cancer Research & Cancer and Blood Disorders, Nemours Children’s Hospital, Wilmington, DE 19803, USA; josh.faust@nemours.org (J.R.F.); darcy.hamill@nemours.org (D.H.); eakolb@nemours.org (E.A.K.); anil.g@nemours.org (A.G.)

**Keywords:** mesothelin, acute myeloid leukemia, immunotherapy

## Abstract

**Simple Summary:**

This review summarizes the current knowledge on mesothelin’s function, its role in cancer, and opportunities for immunotherapeutic targeting of mesothelin. Immunotherapies including monoclonal antibodies, antibody–drug conjugates, chimeric antigen receptor T and NK-cells, targeted alpha therapies, and bispecific T cell engaging molecules are reviewed. We show future directions for mesothelin targeting in hematological malignancies, including acute myeloid leukemia.

**Abstract:**

Modern targeted cancer therapies rely on the overexpression of tumor associated antigens with very little to no expression in normal cell types. Mesothelin is a glycosylphosphatidylinositol-anchored cell surface protein that has been identified in many different tumor types, including lung adenocarcinomas, ovarian carcinomas, and most recently in hematological malignancies, including acute myeloid leukemia (AML). Although the function of mesothelin is widely unknown, interactions with MUC16/CA125 indicate that mesothelin plays a role in the regulation of proliferation, growth, and adhesion signaling. Most research on mesothelin currently focuses on utilizing mesothelin to design targeted cancer therapies such as monoclonal antibodies, antibody–drug conjugates, chimeric antigen receptor T and NK cells, bispecific T cell engaging molecules, and targeted alpha therapies, amongst others. Both in vitro and in vivo studies using different immunotherapeutic modalities in mesothelin-positive AML models highlight the potential impact of this approach as a unique opportunity to treat hard-to-cure AML.

## 1. Introduction

As medicine moves from a traditional framework to a more personalized approach in cancer therapeutics, there is a need to identify tumor-specific antigens. One of the most notable, mesothelin, is a glycosylphosphatidylinositol (GPI)-anchored cell surface glycoprotein overexpressed in many cancers. Mesothelin was first discovered while attempting to identify novel cell surface markers in ovarian cancer by isolating antibodies that are reactive with the surface of these cells. The monoclonal antibody (mAb) K1 was isolated from mice immunized with human ovarian carcinoma cells, OVCAR-3 [1]. When evaluated through immunohistochemistry, this mAb was also found to be reactive with normal mesothelia of the peritoneum, pleura, and pericardium as well as several ovarian carcinoma, cervical cancer, and gastric cancer cell lines. The antigen was subsequently identified using expression cloning from a HeLa cDNA library and named mesothelin to reflect its expression on normal mesothelial cells [2]. 

Given its limited expression in normal tissues and overexpression in several tumor cells, mesothelin presents a desirable target for tumor-specific therapy. In pre-clinical studies using a human epidermoid carcinoma cell line engineered to express mesothelin, a biodistribution assay with radiolabeled mAb K1 showed good tumor localization and specificity for antigen-expressing tumors [3]. Clinical studies using radiolabeled anti-mesothelin antibodies and PET imaging revealed localization to tumors which expressed mesothelin [4]. To date, several immunotherapeutic modalities, most notably, anti-mesothelin immunotoxins, have been successfully implemented for the treatment of mesothelin-expressing solid tumors, including ovarian carcinomas [5], pancreatic adenocarcinoma [6], and malignant mesothelioma [7], while mesothelin-targeting chimeric antigen receptor (CAR) T cells have shown significant anti-tumor effects [8,9,10]. Attempts to find a tumor-associated antigen on AML via genomic data mining endeavors have recently identified mesothelin overexpression [11], presenting the protein as a potential therapeutic opportunity.

## 2. Structure and Function of Mesothelin

Located at 16p13.3, the mesothelin gene, *MSLN*, contains 17 exons. The 2138-base-pair-long *MSLN* cDNA, with an open reading frame of 1884 base pairs, encodes a 622-residue (69 kDa) precursor protein (pre-pro mesothelin) [2]. The N-terminal signal peptide consisting of residues 1–33 is removed after insertion into the membrane. The truncation of C-terminal residues 599–622 is accompanied by the addition of GPI anchor at S598. This precursor protein is cleaved at R295 residing within a putative proteolytic processing site RPRFRR recognized by furin protease. In the absence of furin, however, mesothelin is still able to be processed through different unidentified proteases, as both mutations in the furin cleavage site or absence of activated furin resulted in the expression of mature, processed mesothelin, albeit at lower expression levels [12]. The proteolytic cleavage of pre-pro mesothelin produces a GPI-anchored 303 amino acid residue (40 kDa) fragment (mesothelin) and a 31 kDa secreted fragment (sometimes referred to as megakaryocyte-potentiating factor; MPF) [2,13]. 

**Figure 1:**
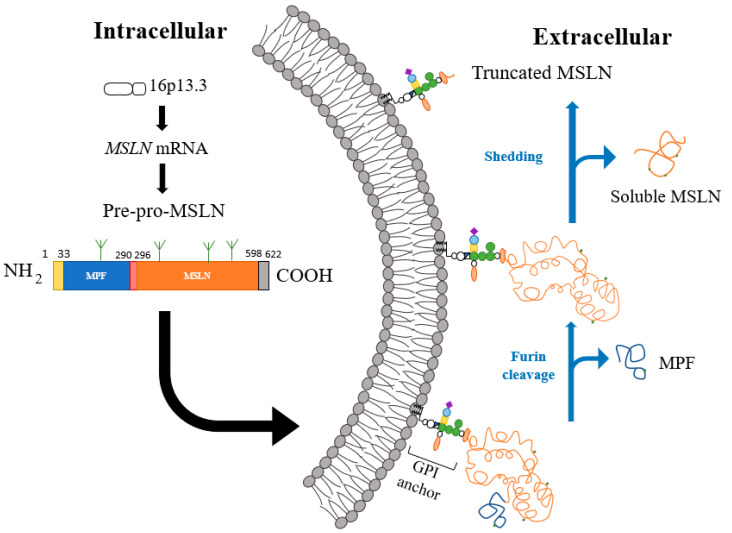
Mesothelin processing produces mature cell-surface mesothelin and megakaryocyte potentiating factor (MPF). The *MSLN* gene is located at 16p13.3 and encodes for the *MSLN* mRNA. The pre-pro-mesothelin consists of a signaling peptide at the N-terminus (yellow), mature MPF (blue), furin cleavage site (red), mature mesothelin (orange), and a GPI-anchor sequence (gray). Along the pre-pro-mesothelin, there are four glycosylation sites (green), three of which exist on the mature mesothelin domain. Once on the surface, mesothelin gets docked to the cell membrane with a glycosylphosphatidylinositol (GPI) anchor. Mesothelin processing continues when furin (or other applicable proteases) cleaves the pre-pro-mesothelin to result in soluble MPF and a mature mesothelin. Finally, mesothelin can undergo membrane shedding and result in a truncated mesothelin protein still left on the cell surface as well as soluble mesothelin.

Mesothelin, like many GPI-anchored proteins, is subjected to shedding to create soluble mesothelin-related peptides (SMRP) that have been used classically as a marker for the detection of malignant mesotheliomas, leaving a small fragment of unshed mesothelin on the cell surface [14,15]. Due to the presence of multiple proteases within the extracellular environment, mesothelin can be cleaved at different extracellular sites depending on the protease [16]. There are seven major cut sites close to the membrane anchor on mesothelin-expressing cells, resulting in the generation of a membrane-bound truncated mesothelin (Figure 1).

Mesothelin precursor, mature protein and MPF belong to a mesothelin superfamily of proteins that also includes hypothetical MPF-like protein, stereocilin and otoancorin, based on sequence homology. Structure prediction programs favor a superhelical structure with ARM-type repeats in all these proteins. This proposed structure model is composed of tandem repeats of helix-turn-helix motifs [17]. No crystal structure has yet been determined for the whole protein, but the structure of an N-terminal fragment (residues 7–64) bound to a Fab fragment of the anti-mesothelin antibody SS1 has been characterized. The N-terminal fragment has a dipole moment and it is proposed that it consists of a loop (residues 7–17), followed by five alpha helices made up of 5, 7, 6, 6, and 13 residues, respectively. Together, these alpha helices, connected by short loops, form a right-handed superhelix [18].

GPI-linked proteins in general are known to be involved in cell signaling+ and adhesion, and as such mesothelin may have a role in these biological processes [19,20]. The exact role of mesothelin under normal physiological conditions remains unknown. Mice with homozygous knockout of *MSLN* had normal development and reproductive capabilities [21], suggesting that mesothelin function is not essential for life. However, the ultrastructure of the mesothelial lining was affected in the *MSLN* knockout mice [22], indicating a possible role for mesothelin in shaping the tumor microenvironment. The growth of cancer cells in the peritoneal cavity of *MSLN* knockout mice was greatly reduced compared to wild-type mice [22,23]. Supplementing mesothelin- or MPF-stimulated growth in *MSLN* knockout mice promoted lung cancer growth [23], demonstrating that mesothelin in the microenvironment promotes cancer growth and metastasis in vivo. The co-culture of ovarian cancer cells with *MSLN* knockout mesothelial cells resulted in a decreased size of multicellular aggregates [22], supporting the role of host mesothelin in tumor cell adhesion, migration, and metastasis [5].

## 3. Mesothelin Binding Partners

The well-known interacting partner of mesothelin is CA125/MUC16, a member of the mucin family of glycoproteins which is expressed in ovarian cancer and malignant mesothelioma [24,25]. CA125/MUC16 was originally used as a biomarker for ovarian cancer given its high expression in tumor cells, and elevated levels in the sera of patients with ovarian cancer [26]. The interaction between CA125/MUC16 and mesothelin mediates heterotypic cell adhesion in vitro and thus has been implicated as a potential mechanism for the peritoneal metastasis of ovarian tumors [24,25]. Kaneko et al. narrowed down the minimal binding site of CA125/MUC16 on mesothelin to residues 296–359 of the N-terminus of cell-surface mesothelin [27]. The binding of CA125/MUC16 to mesothelin downregulates DKK1 (Dickkopf-1, a WNT signaling pathway inhibitor) through the SGK3/FOXO3 signaling pathways, which in turn fuels migration. Blocking CA125/MUC16 and mesothelin binding was able to restore DKK1 levels and prevent ovarian cancer metastasis [28].

Soluble mesothelin binding to surface-anchored mesothelin triggered the expression of matrix metalloproteinase-7 (MMP-7) through the ERK (extracellular-signal-regulated kinase) ½, Akt and JNK (c-Jun N-terminal kinase) signaling pathways [29], resulting in enhanced migration and invasion. MMPs are important regulators of cell behavior, including growth, differentiation, apoptosis, as well as invasion and migration, and their expression and activity have been implicated in many human cancers [30]. Mesothelin overexpression in ovarian cancer cells also had a similar effect and correlated significantly with MMP-7 expression [29]. This could result from trans-interactions between cell surface mesothelin molecules on two adjacent cells or via the action of increased soluble mesothelin. In addition, mesothelin binding to CA125/MUC16 also triggered MMP-7 expression via the p38 MAPK pathway in pancreatic ductal adenocarcinoma [31].

## 4. Role of Mesothelin in Cancers and Signaling

Mesothelin expression has been identified in many solid tumors, most robustly in mesothelioma, epithelial ovarian cancer, and pancreatic adenocarcinoma, but also in lung and uterine malignancies as well as cholangiocarcinoma [32,33,34,35,36]. Available data identify a potential role of mesothelin in tumor cell adhesion, progression, proliferation, survival, and resistance to chemotherapy, although the definitive mechanisms have yet to be fully elucidated [5,25,29,37,38,39,40]. 

A study conducted by Weidemann et al. classified the differential mesothelin expression levels in about 13,000 tumor samples from 122 tumor types through immunohistochemistry. The results show that 54% of tumor types showed at least some weak staining for mesothelin, while 41% of tumor types had at least one strong positive staining sample. The highest prevalence of mesothelin was found in ovarian carcinomas and mesotheliomas, with higher expression being correlated with advanced tumor stages and higher rates of metastasis [41]. In stage IV colorectal cancers, high mesothelin expression was directly correlated with chemoresistance, aggressiveness, and poor prognosis [37]. In contrast, data with pancreatic adenocarcinoma seem to suggest that although mesothelin is commonly expressed, there is no correlation with cancer aggressiveness [42]. This shows that the expression of mesothelin and its relation to prognosis and cancer aggressiveness is cancer specific. 

In solid tumors, the epithelial-to-mesenchymal (EMT) transition is an early key step for tumor invasion and metastasis [43]. Mesothelin expression triggers EMT, with a reduced expression of epithelial markers and increased expression of mesenchymal and stemness markers [44,45]. Lung cancer cells with mesothelin knockdown had reduced anchorage-independent growth, tumor formation, migration, invasion, and metastasis, indicating that mesothelin is a mediator of these important cancer hallmarks. Knockdown of mesothelin also reversed the EMT and diminished stem cell properties [44]. 

**Figure 2:**
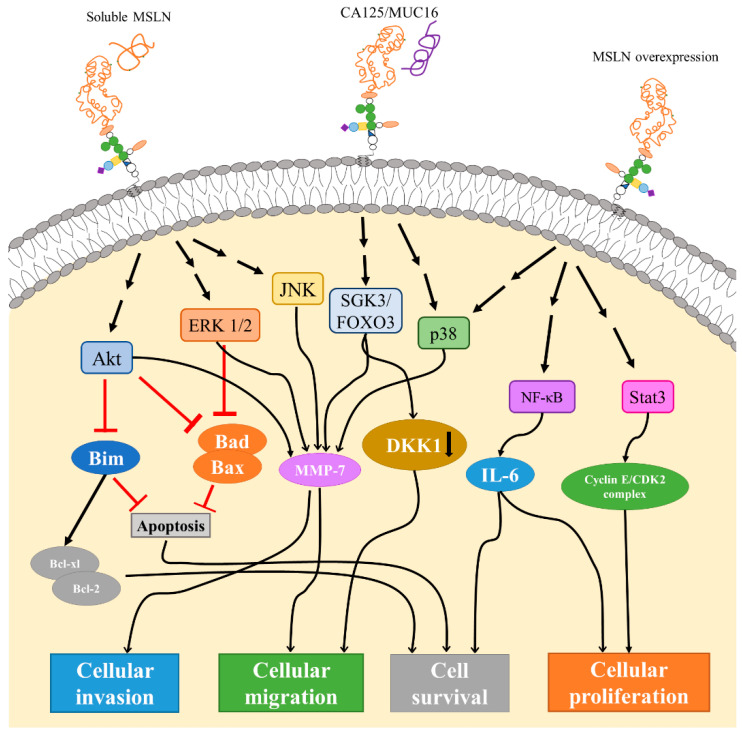
Signaling pathways associated with mesothelin. Through interactions with soluble or cell surface mesothelin, mesothelin can trigger Akt, ERK1/2, and JNK signaling pathways. Downstream effects of Akt and ERK1/2 signaling include inhibition of Bim, Bad, and Bax, which then inhibits apoptosis and/or stimulate Bcl-xl/Bcl-2 leading to cell survival. The Akt, ERK1/2, and JNK pathways are shown to increase expression of matrix metalloprotease 7 (MMP-7), which leads to increased rates of cellular migration and invasion. MMP-7 pathway can also be triggered through SGK3/FOXO3 and p38 pathways. SGK3/FOXO3 signaling has also been shown to cause the downregulation and expression of DKK1, thus increasing cell migration. In general, overexpression of mesothelin also causes p38 signaling activation, as well as NF-kB and STAT3 signaling. NF-kB downstream effect increases expression of IL-6 and contributes to cell survival and proliferation, while STAT3 signaling has been indicated in Cyclin E/CDK2 complex formation, pushing the cell through the cell cycle, and increasing cell proliferation.

Pancreatic cancer cell lines overexpressing mesothelin had significantly increased proliferation and faster cell cycle progression compared to a mesothelin-silenced cell line. The overexpression of mesothelin caused the constitutive activation of STAT3, resulting in the increased expression of cyclin E, cyclin E/cyclin-dependent kinase 2 (CDK2) complex formation, and faster G1-S transition (Figure 2). 

The precise mechanism of STAT3 activation by mesothelin was not determined [38]. Lurie et al. sought to investigate the differential genes that were associated with mesothelin expression in pancreatic ductal adenocarcinoma and found that there are multiple genes that had higher expression in the mesothelin-high group, including genes associated with poor prognosis, such as *KCNN4*, *TNK3*, and *MUC1*. Additionally, retinoic acid receptor gamma (RARγ) and AKT were regulated by mesothelin as the increased expression of mesothelin resulted in a higher expression of both. Interestingly, the overexpression of mesothelin in the mesothelin-low group did not result in higher AKT and RARγ levels, suggesting that mesothelin is necessary, but not sufficient for activation [46].

Mesothelin overexpression in pancreatic cancer cells leads to the constitutive activation of NF-κB, thereby increasing the production of IL-6 and enhanced tumor cell proliferation and survival via auto/paracrine IL-6/sIL-6R trans-signaling. Mesothelin-silenced cells had decreased levels of IL-6 and reduced cell proliferation. [39]. The expression of mesothelin stimulates MAPK/ERK signaling pathways that, in turn, inhibit pro-apoptotic family proteins, such as Bim, Bad and Bax. Additionally, mesothelin stimulates PI3K/Akt signaling pathway to also inhibit Bad and Bax, while promoting anti-apoptotic proteins, Bcl-xl and Bcl-2, overall resulting in the inhibition of apoptosis and increased cell survival [47]. Mesothelin silencing by siRNA and microRNA decreased cell viability and invasiveness in multiple cancer types by reduced ERK1 and PI3K/AKT signaling [45], further confirming that mesothelin overexpression activates pro-survival signaling.

The treatment of ovarian cancer cells with metformin, an anti-diabetic drug that also shows anti-cancer properties, reduced mesothelin expression. The downregulation of mesothelin decreased IL-6/STAT3 signaling, leading to the inhibition of cell growth and migration and increased rates of apoptosis [48]. 

Since mesothelin is a GPI-anchored protein that lacks both a transmembrane domain and an intracellular domain, the transduction of signals from the extracellular interactions into the cell and the intracellular signaling components is widely unknown and unstudied. It is thought that GPI-anchored proteins transduce cellular signals through the association of the anchor with transmembrane proteins that play a role in intracellular signaling, such as receptor tyrosine kinase and integrins or via interactions with adaptor proteins. Additionally, GPI-anchored proteins often associate in lipid rafts, thus making it likely that mesothelin associates with a transmembrane protein in this microdomain [49,50].

## 5. Regulation of Mesothelin Expression

A 20-base-pair promoter region, Canscript, containing an SP1-like element and MCAT element, has been identified as responsible for mesothelin expression in certain cancers [51]. The reporter with the full Canscript region was found to be 20 times more active than a matched reporter which lacked Canscript in mesothelin over-expressing pancreatic cancer cells. The MCAT element within the Canscript region was the binding site for the transcription enhancer factor (TEF)-1, implicating TEF’s role in the regulation of mesothelin expression [51]. *MSLN* gene transcripts exist in multiple variants with differential splicing in regions spanning exons 15–17. The minor splice variants resulting from incomplete processing were inconsequential in ovarian and pancreatic carcinoma cell lines [52].

At the epigenetic level, *MSLN* expression is regulated mainly via promoter methylation. A pancreatic cell line resistant to mesothelin-targeting drugs was determined to have a 5-fold decrease in the cell-surface expression of mesothelin comparative to a sensitive cell line. This downregulation of *MSLN* was caused by the methylation of the promoter, and DNA methyltransferase inhibitor, 5-azacytidine, was able to restore *MSLN* expression [53]. The hypomethylation of the promoter was noted in mesothelin-expressing pancreatic cancer specimens, and the treatment of a non-expressing pancreatic cancer cell line with demethylating agents could induce the expression of mesothelin, suggesting that epigenetic mechanisms regulate mesothelin expression in this cell type [54]. Interestingly, *MSLN* promoter hypomethylation was seen in mesothelioma but not associated with mesothelin expression [55], indicating that there may be another layer of post-transcriptional regulation of mesothelin expression in mesothelioma.

An investigation of microRNA through next-generation sequencing revealed miR-21 as a regulator of mesothelin expression, where the inhibition of miR-21 restored mesothelin expression in a previously non-expressing model [56]. In pancreatic cancer, miR-198 was determined to be a key player in a regulatory feedback loop with mesothelin, wherein mesothelin represses miR-198 through OCT-2 induction mediated by NF-κB [57].

Mesothelin expression is regulated by different Wnt proteins, which have been previously implicated in tumorigenesis. Mesothelin was specifically upregulated by Wnt-1 and those tumors with constitutive Wnt signaling pathway activation also had high expression of mesothelin [45,58]. More studies are necessary to determine the mechanism of mesothelin regulation by Wnt/beta-catenin signaling. 

## 6. Mesothelin Targeting Therapies

Mesothelin is considered a differentiation antigen and represents a desirable candidate for targeted therapy given its limited expression in normal tissues and overexpression in several malignancies. Furthermore, the surface expression of mesothelin allows for the specific targeting of malignant cells [59]. The results of several ongoing clinical trials of immunotherapy agents directed against mesothelin (Table 1) have been shown to be safe, without excessive toxicity. These agents include immunotoxin therapy, monoclonal antibodies, vaccine-directed therapy, antibody–drug conjugates, chimeric antigen receptor T cells, chimeric antigen receptor NK cells, T cell receptor-like agents, and alpha targeted therapies [60].

The response to immunotherapy agents is often restricted by the interactions with immune checkpoint proteins. Programmed cell death protein 1 (PD-1) is one such extracellular protein expressed on activated T cells that helps modulate the T cell response. Programmed cell death ligand 1 (PD-L1) is expressed on antigen-presenting cells (APCs) and the binding of PD-1 and PD-L1 is considered coinhibitory as it can reduce T cell activity through reduction in cytokine secretion [69]. The expression of PD-L1 on tumor cells limits the efficacy of immune cells, as these cells do not recognize the tumor cell as their ideal target. Therefore, immune checkpoint blockade therapies are used in combination with immunotherapeutics to overcome the immune evasive response. 

### 6.1. Immunotoxins and Monoclonal Antibodies

SS1P is an anti-mesothelin immunotoxin, an antibody-based therapy with a bacterial toxin payload. This recombinant protein consists of a murine Fv fragment linked to a *Pseudomonas* exotoxin A payload [70]. Although the toxin was well tolerated and exhibited significant anti-tumor activity [59], the efficacy was limited by the development of neutralizing antibodies. This limitation was overcome by combining SS1P with chemotherapy agents that suppress the host immune system [71]. LMB-100 is a second-generation immunotoxin comprising a humanized anti-mesothelin fragment and a modified *Pseudomonas* exotoxin A payload which was designed to be less immunogenic [72]. LMB-100 has been similarly limited by the development of anti-drug antibodies [73,74]. In mesothelioma, the combination of LMB100 and anti-PD-1 antibody therapy has enhanced efficacy comparative to solo therapies [75]. Both modalities continue to be studied in combination with chemotherapy in mesothelin-expressing solid tumors.

Amatuximab (originally called MORAb-009) is a monoclonal antibody which targets mesothelin, thereby disrupting cell adhesion and initiating antibody-dependent cytotoxicity [76]. The anti-tumor effect and maximum tolerated dose have been established in completed phase I and II clinical trials in combination with chemotherapy [77,78]. In vitro studies indicate that amatuximab inhibits mesothelin interaction with CA125/MUC16 and such a blockage reduces the cancer’s ability to metastasize and invade into other tissues. Additionally, amatuximab treatment downregulated cancer stem cell markers, such as CD44, c-MET, and ALDH1 in pancreatic cancer cells [79]. Amatuximab treatment increased sensitivity to gemcitabine and reduced the expression of c-Met and AKT in the liver, accompanied by decreased rates of pancreatic cancer cell metastasis [79,80].

### 6.2. Vaccines

GVAX is a granulocyte–macrophage colony-stimulating factor (GM-CSF) tumor cell vaccine which expresses multiple antigens and induces anti-tumor immune responses by cross-priming mechanisms to recruit antigen-presenting cells [81]. Mesothelin was identified as a target of T cells in patients treated with GVAX, initiating the development of a *Listeria monocytogenes* vaccine (CRS-207), which expresses tumor-associated antigen mesothelin. CRS-207 secretes mesothelin in the cytosol of infected antigen-presenting cells, which is processed and presented by major histocompatibility complex (MHC), thereby stimulating an immune response [82,83]. A phase II study using CRS-207 in combination with GVAX and conventional chemotherapy for patients with previously treated metastatic pancreatic adenocarcinoma improved overall survival [84]; however, a follow-up phase IIb study showed similar survival as chemotherapy [85]. A phase I study of CRS-207 in addition to conventional chemotherapy for the treatment of patients with malignant pleural mesothelioma found that it was well tolerated, with few adverse events. They reported reduced tumor sizes and an improvement in survival, including one complete response [86].

### 6.3. Antibody–Drug Conjugates

Anetumab ravtansine is an antibody–drug conjugate (ADC) comprised of a humanized anti-mesothelin antibody conjugated to DM4 (a maytansinoid tubulin inhibitor), which showed potent killing of tumor cells expressing mesothelin [87]. The drug binds specifically to mesothelin-expressing tumor cells and releases its cytotoxic payload following internalization. Phase I trials in patients with advanced solid tumors suggested that anetumab ravtansine can be safely given to patients and supported its anti-tumor efficacy [88]. Additional phase I and II trials are ongoing. Preclinical studies showed an improved anti-tumor effect when used in combination with standard chemotherapy in ovarian cancer models [89]. 

ADC DMOT4039A, a humanized anti-mesothelin monoclonal antibody conjugated to monomethyl auristatin E (MMAE), which has anti-mitotic effects, was found to be safe in a phase I clinical trial [90].

Another immunoconjugate, BMS-986148, conjugated to tubulysin, which disrupts microtubule assembly and induced apoptosis, was evaluated in solid tumors. Phase 1/2a studies of BMS-986148 treatment alone or in combination to nivolumab, a PD-1 inhibitor, was concluded to have manageable safety as well as clinical activities, warranting further clinical trials in combination with other checkpoint inhibitors [91]. 

### 6.4. Chimeric Antigen Receptor T Cells and T Cell Receptor Fusion Constructs 

Chimeric Antigen Receptor (CAR) T cells are autologous patient T cells which are genetically modified to target a cancer-specific protein. When these cells bind to their target, they become activated, proliferate and mediate cytotoxic effects [92]. CAR-meso T cells consist of an anti-mesothelin scFv fused to TCRzeta signaling and costimulatory domains, allowing for specific binding to mesothelin expressing cells and subsequent cytotoxic response [93,94]. Preclinical studies of mesothelin targeting CAR-T cells have shown significant tumor reduction and are currently under clinical evaluation by many groups [93,95]. Phase I studies have shown that mesothelin targeting CAR-T cells are well tolerated with minimal on-target off-tumor effects but showed limited clinical activity [65]. There are many ongoing trials investigating mesothelin-directed CAR-T cells in solid tumors [96]. 

Most recently, novel T cell engineering platforms which target mesothelin have been investigated. T cell receptor fusion constructs (TRuCs) target tumor cells independent of MHC, resulting in increased T cell activation. Preclinical studies with mesothelin-directed TRuCs have shown robust anti-tumor activity, with faster rates of accumulation in mesothelin-expressing tumors, lower levels of cytokines, increased levels of chemokine receptors, and long-term functional persistence [97].

#### CAR-T Cell Alterations

CAR-T cells are conventionally designed against single antigens of interest; however, Tandem CAR-T cells targeting two antigens may be more effective and have higher anti-tumor effects than single antigen targeting CAR-Ts. These Tandem CAR-Ts allow for simple Boolean logic gates of “AND”, “OR”, or “NOT”, as they can recognize multiple antigens, and have been used in hematological malignancies, including acute lymphoblastic leukemia to simultaneously target CD19 and CD20 [98,99]. Tandem CAR-Ts targeting cancer-specific upregulated molecules mesothelin and folate receptor 1 (FOLR1) with secretory activity of IL-12 had higher infiltration and persistence comparative to anti-mesothelin CAR-Ts, and the secretion of IL-12 enhanced therapeutic effects and reduced tumor antigen escape in ovarian cancer [100]. These data suggest that the identification of multiple tumor-associated antigens would improve the results of CAR-T cells by applying Boolean logic for the recognition of multiple targets to reduce antigen escape. 

The silencing of PD-1 on CAR-T cells with the use of shRNA resulted in increased efficacy and an enhanced anti-tumor effect on several mesothelin-expressing cancers [101]. The success of PD-1 silencing in CAR-T cell therapy opens the door to the gene silencing or activation of other genes in these cells to improve anti-tumor effects. Another antigen of interest with CAR-T cells is Tim3 (T cell immunoglobulin domain and mucin domain 3), which is an immune checkpoint receptor that helps regulate T cell response in the tumor microenvironment. Tim3 plays a role in immunosuppression and T cell death. Blocking Tim3 function reduced immunosuppression, reduced regulatory T cells, and increased IFN-γ production from T cells [102,103]. Blocking Tim3 expression on mesothelin CAR-T cells through shRNA had improved cytotoxicity effects, increased cytokine production, and higher proliferation capacity compared to their Tim3+ counterparts [104]. These two studies present the possibility of customizing CAR-T cells to overcome mechanisms that inhibit their function within the tumor microenvironment and will need further investigation to identify and silence other immunosuppressive targets.

Another factor that decreases the efficacy of CAR-T cells is the production of adenosine in the tumor microenvironment. The overproduction of adenosine from tumor cells facilitates immunosuppression through the binding of the adenosine 2a receptor (A2aR). The knockdown of A2aR through shRNA in CAR-T cells increased the anti-tumor ability of the CAR-T cells both in vivo and in vitro. In xenograft models, the A2aR-disrupted anti-mesothelin CAR-T cells decreased the tumor burden compared to unmodified T cells and anti-mesothelin CAR-T cells [105]. Expression of anti-mesothelin CAR-T cells with cell chemokine receptors CCR2b and CCR4 revealed enhanced migration of CAR-Ts in vitro and displayed high levels of cytotoxicity and increased levels of IL-2, IFN-γ, and TNF-α, revealing CCR2b as the superior chemokine receptor in NSCLC in vivo studies [106].

### 6.5. Chimeric Antigen Receptor Natural Killer Cells

Similar to CAR-T cells, natural killer (NK) cells can also express chimeric antigen receptors and are possibly more effective than CAR-T cells due to their increased ability to recognize and kill tumor cells to result in tumor-specific killing. In a study on ovarian cancer, CAR-NK cells were designed to recognize mesothelin, and were effective in eliminating mesothelin+ cancer cells through increased cytokine secretion compared to the parental NK cell treatment [107]. In an in vitro study using anti-mesothelin NK cells against gastric cancer, mesothelin-NK cells were more selective in killing mesothelin+ tumors and secreting cytokines comparative to transduced NK cells and were able to prolong survival rates of patient-derived xenograft (PDX) mice in vivo [108].

### 6.6. Bispecific T Cell-Engaging Molecules

Bispecific T cell engagers (BiTEs) are small fusion proteins consisting of two single chain variable fragments (scFvs): one which targets an effector cell (most commonly CD3 on T cells) and another which targets the tumor antigen. These bispecific antibodies redirect the cytotoxic effects of T cells toward specific tumor cells [109]. While the use of BiTEs has been most notable in hematologic malignancies and B cell acute leukemias in particular, they have been developed for a number of different tumor antigens. Research into the use of BiTEs which target mesothelin are currently underway in triple-negative breast cancer, pancreatic ductal adenocarcinoma, lung cancer, and other solid tumors [110,111]. 

One drawback to using BiTEs in vivo is their short half-life of a few hours, thereby requiring administration via intravenous infusions. To overcome this limitation, Suurs et al. designed an anti-mesothelin/anti-CD3 BiTE that also had an Fc-domain that increased the half-life and required only intermittent dosing of BiTEs. PET imaging in BALB/c mice revealed the tumor-tissue specific uptake of the BiTE molecule and a half-life of about 63 h [112]. A similar bispecific antibody in the IgG format with bivalency for mesothelin in addition to binding CD3 showed higher efficacy than a corresponding antibody with monovalent mesothelin binding capacity [113]. A trispecific T cell-activating construct, HPN536, has been used to treat mesothelin-expressing solid tumors. In addition to binding to CD3 and mesothelin, HPN536 binds serum albumin to increase the plasma half-life of the molecule. In in vivo studies of cynomolgus monkeys, HPN536 was well tolerated and exhibited mesothelin-dependent pharmacokinetics, which led to a phase I clinical trial (NCT03872206) using HPN536 against solid tumors [114].

### 6.7. Targeted Alpha Therapies

Targeted alpha therapies (TAT) represent a new emerging class of targeted cancer therapies that use high-energy emissions from alpha particles to elicit permanent double-stranded breaks in DNA, ultimately resulting in cell death [115]. Targeted thorium-227 conjugates (TTC) represent a subtype of TATs that consist of a covalently attached 3,2-HOPO chelator to a specified antibody to ensure the delivery of thorium-227, an alpha particle emitter, to mesothelin-expressing cells. The specific mesothelin TTC, BAY 2287411 in mesothelioma, ovarian, and breast cancers, among others, showed anti-tumor potency both in vitro and in vivo in PDX models. The cellular response of BAY 2287411 included increased DNA double-stranded breaks, oxidative stress, and apoptotic markers. The results from this study support the transition of BAY 2287411 to a phase I clinical trial to treat ovarian and mesothelioma cancers [67]. A combination therapy of BAY 2287411 with DNA damage response inhibitors results in a synergistic effect by sensitizing the cells to DNA damage [116]. Targeted alpha therapies remain as one of the least investigated targeted therapeutic options when treating mesothelin-positive tumors. 

### 6.8. Impact of Mesothelin Shedding on Mesothelin-Targeting Therapies

As previously noted, many GPI-anchored proteins undergo routine extracellular shedding, to which mesothelin is no exception. Shed mesothelin could decrease the efficacy of targeted therapies, resulting in an on-target, off-tumor response of the drugs interacting with soluble mesothelin. The most notable sheddase involved in mesothelin shedding is TACE/ADAM17; however, many members of the ADAM, MMP, and BACE protease families are able to shed mesothelin from the cell surface. Inhibiting the shedding of mesothelin with drugs that limit functionality of sheddases has shown promising results in vitro by increasing drug efficacy and cell surface mesothelin [16,61]. The identification of an epitope on the fragmented mesothelin that remains unshed on the surface of the membrane would be more effective in targeting mesothelin.

## 7. Role of Mesothelin in Hematological Malignancies

The overexpression of mesothelin has been well described in several solid tumors; however, there are limited data regarding its expression in hematologic malignancies. Mesothelin expression was lacking in all 442 tissue microarray specimens, representing different types of lymphoma. This study did not evaluate samples from other hematological malignancies [41]. In 2006, Steinbach et al. identified MSLN as one of the seven genes overexpressed in pediatric AML compared to normal bone marrow [117]. Further investigation detected mesothelin protein expression in a small number of primary pediatric AML samples [118]. Recently, massive sequencing efforts in pediatric AML under the direction of Children’s oncology group and NCI (TARGET initiative) identified mesothelin to be expressed in greater than a third (36%) of AML samples [11]. Mesothelin expression was quantified and subsequently confirmed using quantitative RT-PCR and flow cytometry. Data from TCGA and BEAT databases showed that only about 14% of adult AML samples expressed mesothelin. Among the various cytogenetic subtypes of pediatric AML, core binding factor AML (comprising inv(16), t(8;21)), and *KMT2A* rearranged AML comprised 49% and 40% of samples with mesothelin positivity, respectively. 

How mesothelin expression is triggered in AML is not known. In pediatric AML patients, genomic characterization revealed that mesothelin expression is inversely correlated with *MSLN* promoter hypermethylation, consistent with previously described data from solid tumor studies. AML samples with high promoter methylation were characterized as low mesothelin expressers via flow cytometry [11]. 

**Figure 3:**
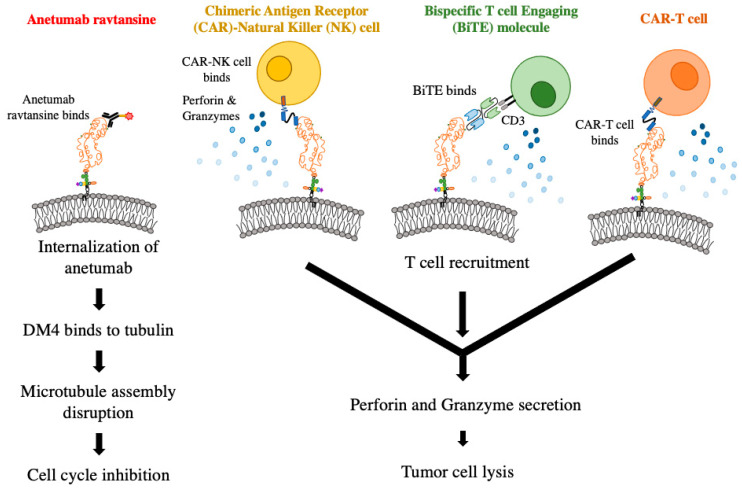
Mesothelin-targeted therapies in preclinical development for pediatric AML. Anetumab ravtansine is an antibody–drug conjugate (ADC) that is an scFv for mesothelin attached to DM4 (red star), a tubulin inhibitor, via a linker (yellow). Once bound to mesothelin, DM4 is internalized and binds to tubulin, which disrupts microtubule assembly and cell cycle is inhibited, which results in cell death. Chimeric antigen receptor (CAR) therapy uses a variety of host immune cells to elicit a tumor-targeted response using a construct designed to recognize a target antigen. In AML, CAR-NK cells (yellow), and CAR T cells (orange) have been investigated. Upon the recognition of the target antigen, NK and T cells activate and elicit a killing mechanism through the secretion of perforin and granzymes (blue), resulting in tumor cell lysis. Bispecific T cell-engaging molecules (BiTEs) work mechanistically by recruiting T cells to tumor cells by engaging with a tumor-associated antigen (shown in blue), while the other side of the molecule engages with a CD3 on a T cell (shown in green). Once bound, the T cell becomes activated and kills through the same mechanisms at the CAR cell lysis.

Preclinical studies to evaluate the efficacy of mesothelin-targeted therapies such as immunotoxin, ADC, BiTEs, CAR-T cells, and CAR-NK cells in pediatric AML have been conducted (Figure 3). An early study using recombinant anti-mesothelin immunotoxin SS1(dsFv)PE38 in vitro failed to produce a cytotoxic effect. The authors speculated that other mesothelin-targeted approaches could be more beneficial [118]. Accordingly, both in vitro and in vivo evaluations of ADC anteumab ravtansine in AML samples resulted in the selective killing of mesothelin-expressing cell lines. Mice xenografted with mesothelin+ samples survived longer when treated with the ADC compared to the isotype drug conjugate control and mesothelin-negative samples exposed to either treatment. Another MSLN-ADC, DGN462, bearing a DNA alkylating agent, also showed preclinical efficacy in pediatric AML [11]. The combination of the ADC treatment with traditional chemotherapy regimen of daunorubicin and cytarabine led to higher survival rates compared to any treatment alone, suggesting a synergistic effect [119]. A phase I clinical trial for anetumab ravtansine is currently under development by the Children’s Oncology Group for mesothelin+ pediatric AML patients in second relapse [120].

Mesothelin-targeting BiTEs were found to be highly effective at inducing complete remission in two PDX models of *KMT2A* rearranged AML. Because these studies were performed in immunodeficient mice, human T cells were injected to act as effector cells. BiTE molecules in the IgG format with a longer in vivo half-life were able to suppress the extramedullary tumor masses that are commonly observed in MV4;11 xenografted mice [63]. CAR-T cells targeting mesothelin were efficacious in controlling tumor burden by eradicating leukemia stem cells [61]. Most recently, work has been conducted using anti-mesothelin CAR NK-92 cells in AML samples. In vitro and in vivo studies revealed that these CAR NK-92 cells were effective at specifically targeting mesothelin+ AML samples while not killing mesothelin-negative samples [62]. These different modalities that target mesothelin provide effective targeted therapeutic options for mesothelin+ AML. 

What is the role of mesothelin in AML? Of important clinical significance, in a review of patients with core binding factor AML, which traditionally confers a more favorable risk stratification, those with concurrent mesothelin expression had a statistically significant higher risk of relapse and worse disease-free survival [121]. Mesothelin expression was significantly associated with the presence of extramedullary disease, which may portend poor survival in AML patients [63]. Interestingly, mesothelin promoted leukemia growth and was also associated with extramedullary disease in AML xenograft models [122]. Mesothelin expression in leukemia stem cells suggests that similar to solid tumors, mesothelin may play a role in leukemia progression and relapse [61]. Further studies are necessary to shed light on the role of mesothelin in hematological malignancies.

In summary, recent findings of mesothelin overexpression in pediatric AML suggest this may be a useful target for therapy. Given the overall poor outcomes for children with AML, further studies to explore the use of mesothelin-directed immunotherapy are warranted. Pre-clinical studies utilizing anti-mesothelin CAR-T cells, BiTEs, and anetumab ravtansine against mesothelin-expressing AML exhibit tumor-specific killing of leukemia cells, and the ability to reduce leukemic burden in vivo. These studies of monotherapies as well as combination therapies with chemotherapy and/or immune checkpoint blockade inhibitors present a strong case for transition into clinical studies. 

## 8. Conclusions

Mesothelin is a highly desirable target for tumor-specific immunotherapy given its overexpression in tumor cells and relative paucity in normal healthy cells. Currently, research seems to focus on the use of mesothelin to produce promising targeted therapy approaches, and to match the needs of the patient population and the tumor microenvironment specific to each cancer type. Many different targeted approaches allow for slight alterations that can improve and make the therapies more targeted in relation to difficulties, such as silencing immunosuppressive molecules. There still is a need, however, to identify the function of mesothelin, and other key characteristics of mesothelin, such as the unshed epitope to increase the therapeutic potential. While mesothelin has been considered a target for solid tumors, recent findings of mesothelin overexpression in AML point towards a further evaluation of the expression of this marker in other hematological malignancies. The absence of mesothelin on normal and healthy hematopoietic cells is of particular importance, as currently available immunotherapies for the treatment of AML are associated with hematopoietic toxicities.

## Figures and Tables

**Table 1:** Current pre-clinical and clinical studies targeting mesothelin (MSLN) in solid tumors and hematological malignancies.

Phase	Tumor Type	Mechanism	NCT Number	Reference
Pre-clinical	MSLN+ AML	anti-MSLN CAR-T cells	N/A	[61]
Pre-clinical	MSLN+ AML	anti-MSLN NK cells	N/A	[62]
Pre-clinical	MSLN+ AML	BiTEs	N/A	[63]
Pre-clinical	MSLN+ AML	Anetumab ravtansine	N/A	[11]
Early Phase 1	Ovarian cancer	anti-MSLN CAR-T cells, fludarabine and cyclophosphamide	NCT03814447	
Phase 1	Refractory malignant solid neoplasm	TCR-T cells, cyclophosphamide and fludarabine	NCT04809766	
Phase 1	Solid tumors	anti-MSLN CAR-T cells	NCT03545815	[64]
Phase 1	Lung adenocarcinoma, ovarian cancer, peritoneal carcinoma	anti-MSLN CAR-T cells	NCT03054298	[65]
Phase 1	Malignant pleural mesothelioma	anti-MSLN CAR-T cells and cyclophosphamide	NCT04577326	
Phase 1	MSLN+ neoplasms, epithelioid mesothelioma, cholangiocarcinoma, pancreatic adenocarcinoma	LMB-100 and Tofacitinib	NCT04034238	[66]
Phase 1	Mesothelioma	LMB-100 and Ipilimumab	NCT04840615	
Phase 1	Advanced recurrent epithelioid mesothelioma, serous ovarian cancer, metastatic or locally advanced pancreatic ductal adenocarcinoma	BAY2287411 (MSLN-TTC)	NCT03507452	[67]
Phase 1, 2	Advanced MSLN+ tumors	HPN536	NCT03872206	
Phase 1, 2	Pancreatic adenocarcinoma	Anetumab ravtansine, gemcitabine hydrochloride, Ipilimumab and Nivolumab	NCT03816358	
Phase 2	Fallopian tube endometrioid adenocarcinoma, high grade fallopian tube and ovarian serous adenocarcinoma	Anetumab ravtansine, Bevacizumab and paclitaxel	NCT03587311	
Phase 2	Non-small cell lung cancer	LMB-100 and Pembrolizumab	NCT04027946	
Completed	Malignant. Mesothelioma	SS1P, pentostatin, and cyclophosphamide	NCT01362790	
Completed	Pancreatic cancer	GVAX and CRS-207	NCT02243371	[68]
